# Silverleaf Nightshade ( *Solanum elaeagnifolium* ) Undergoes Guild‐Specific Transcriptomic Reprogramming Against Chewing Versus Piercing Sucking Herbivory

**DOI:** 10.1111/ppl.71128

**Published:** 2026-09-24

**Authors:** Arunsaikumar Karrem, Matthew Terry, Jagdish Jaba, Rupesh Kariyat

**Affiliations:** ^1^ Department of Entomology and Plant Pathology University of Arkansas Fayetteville Arkansas USA; ^2^ School of Integrative Biological and Chemical Sciences The University of Texas Rio Grande Valley Edinburg Texas USA; ^3^ International Crops Research Institute for the Semi‐Arid Tropics Hyderabad Telangana India

**Keywords:** feeding guilds, hub genes, *Solanum elaeagnifolium*, transcriptomics, WGCNA

## Abstract

*Solanum elaeagnifolium* (silverleaf nightshade, SLN) is a globally invasive perennial weed with reproductive plasticity and complex defence systems that significantly impact crop yields by invading fields of staple, commercial and ornamental crops. Despite being a host to multiple insect herbivores, the molecular programming enabling its rapid adaptation to defend against diverse feeding guilds remains poorly resolved. Here, we report a comprehensive de novo transcriptomic assembly and comparative analysis of *S. elaeagnifolium* challenged by two distinct herbivores: the piercing‐sucking generalist cowpea aphid, *Aphis craccivora* and the chewing specialist tobacco hornworm, *Manduca sexta* . Differential gene expression analysis revealed a massive difference in the scale of the plant's response; aphid infestation resulted in 14,799 differentially expressed genes (DEGs), whereas hornworm feeding triggered a more localized response of 4235 DEGs. Guild‐specific co‐expression networks and transcription‐factor enrichment (907 DEGs in aphid‐challenged versus 196 in hornworm‐challenged) point to two distinct regulatory responses. This reprogramming strategy was characterized by enriched KEGG pathways for sphingolipid metabolism and MAPK signalling in aphids. Conversely, hornworm infestation resulted in significant enrichment of cell wall organization, pectin catabolism and callose synthesis. Functional enrichment of network hub genes further revealed aphid‐specific chromatin remodelling and DNA repair activity, while hornworm hub genes were associated with ribosome biogenesis and auxin‐responsive GH3‐family enzymes implicated in jasmonate conjugation. These contrasting findings indicate that SLN does not deploy a generic wound response but instead reallocates transcriptional resources according to feeding mode. This guild‐specific defensive plasticity likely underlies part of the exceptional invasive success of SLN across diverse environments.

## 1 Introduction

Invasive weeds pose a serious threat to global biodiversity and agricultural productivity, often outcompeting native flora through enhanced fitness and defense traits (Richardson and Pyšek 2006; Petanidou et al. 2018; Chavana et al. 2021). Among the most problematic of these weeds is *Solanum elaeagnifolium* Cavanilles (Silverleaf Nightshade, SLN), a perennial noxious weed native to the southwestern United States and northern Mexico that has now invaded nearly all continents (Travlos 2013; Tsaballa et al. 2015; Chavana et al. 2021; Tataridas et al. 2023). In the United States alone, invasive species collectively cause an estimated $120 billion in annual economic damages (Pimentel et al. 2005; Kasper et al. 2021), with *S. elaeagnifolium* contributing through direct crop yield losses via resource competition, reduction of hay quality in infested fields and livestock toxicity caused by its glycoalkaloid compounds (Boyd et al. 1984; Kasper et al. 2021). The weed is listed as a noxious species in 21 US states, competes with crops by growing deeper roots than associated agricultural species, and is exceptionally difficult to eradicate due to its extensive root system, herbicide resistance and ability to regenerate from root fragments (Boyd et al. 1984; Chavana et al. 2021).

SLN causes significant economic losses in major crops such as cotton, wheat, tomato, and its resilience is bolstered by a dual reproductive strategy with both sexual seed production and aggressive asexual propagation through underground rhizomes (Cuthbertson 1976; Brunel 2011; Tataridas et al. 2023). Furthermore, its ability to thrive under severe environmental stressors, particularly anthropogenic disturbances like continuous mowing and deep soil tillage, has led to it being called a “superweed” (Chavana et al. 2021; Vasquez et al. 2024). Recent studies indicate that while mowing reduces overall fruit set, it acts as a selective pressure that produces heavier seeds with faster germination rates and enhanced foliar and floral defenses, facilitating the weed to rapidly return to disturbed environments (Chavana et al. 2021; Vasquez et al. 2024). As a member of the Solanaceae family, SLN is part of a complex trophic network involving diverse insect herbivores that use distinct feeding strategies (Watts and Kariyat 2021).

SLN possesses an arsenal of constitutive and induced defenses, including glandular and non‐glandular stellate trichomes, spines and secondary metabolites like terpenes (e.g., (E)‐caryophyllene; Tsaballa et al. 2015) and polyphenol oxidase (PPO; Vasquez et al. 2024), which are mobilized specifically to deter stressors. SLN has also been found to undergo strategic trade‐offs in defense allocation when subjected to mechanical disturbances. Continuous mowing, which functions as a form of repetitive mechanical wounding, significantly impacts the plant's resistance to different herbivore guilds (Mithöfer et al. 2005; Engelberth and Engelberth 2019). Research has demonstrated that mowed plants exhibit enhanced resistance to chewing herbivores; for instance, *M. sexta* caterpillars show significantly lower mass gain on seedlings derived from mowed parents (Chavana et al. 2021). Conversely, this same disturbance increases susceptibility to piercing‐sucking insects, as evidenced by the significantly higher establishment and population growth of *A. craccivora* on mowed seedlings (Chavana et al. 2021). In other plant systems, such patterns have been attributed to negative cross‐talk between the jasmonic acid (JA) and salicylic acid (SA) pathways, where wound‐related JA defenses suppress SA‐mediated resistance, leaving the plant vulnerable to phloem‐feeding insects (Traw et al. 2003; Bostock 2005; Schweiger et al. 2014; Costarelli et al. 2020; Liu et al. 2025). However, whether or not? this mechanism activates in *S. elaeagnifolium* needs direct experimental investigations.

While the ecological phenotypes of differential herbivore performance and defense response under mowing are well‐documented in SLN (Chavana et al. 2021; Kasper et al. 2021; Vasquez et al. 2024), the molecular processes governing these divergent responses remain unknown. Although a de novo transcriptome for SLN has been developed, identifying over 75,000 unigenes including those for terpene biosynthesis (e.g., *DXS2*, *TPSs*) and wounding markers (e.g., *AOC*; Tsaballa et al. 2015), a comprehensive map of how the transcriptome is reprogrammed under the dual pressure of guild‐specific herbivory remains unexplored. Understanding how this superweed crosstalks at the molecular level is essential to unravel the trade‐offs that allow this weed to thrive despite intensive management (Roberts and Florentine 2022; Tataridas et al. 2022).

To address this, the current study focuses on two representative herbivores from different feeding guilds: the tobacco hornworm, *Manduca sexta* , a biting‐chewing specialist and the cowpea aphid, *Aphis craccivora*, a piercing‐sucking generalist. Evidence suggests that these guilds trigger different physiological responses in the host, chewing herbivores like *M. sexta* typically induce the JA signaling pathway in well‐characterized model systems (Halitschke et al. 2001; Halitschke and Baldwin 2003), which regulates structural defenses such as trichomes and spines (Escobar‐Bravo et al. 2017), whereas sucking herbivores like aphids generally trigger the SA pathway (Mohase and van der Westhuizen 2002; Smith et al. 2009; Escobar‐Bravo et al. 2017). The primary objective of this study is to utilize de novo transcriptomics to map the molecular logic of guild‐specific defenses in *S. elaeagnifolium* . By comparing the transcriptional profiles of SLN challenged with *M. sexta* and *A. craccivora* , we aim to identify the specific hub genes and divergent metabolic pathways that underlie these resistance trade‐offs. Mapping these transcriptional programs will provide critical insights into the evolutionary ecology of this noxious weed and facilitate the development of more effective, ecologically informed management strategies.

## 2 Materials and Methods

### 2.1 Plant Material and Insect Infestation

SLN seeds were collected from greenhouse grown plants, initially collected from a field population in the McAllen‐Edinburg area of Rio Grande Valley, Texas, USA (Chavana et al. 2021). At maturity, five pods each were collected from randomly selected five maternal plants and were used to extract seeds. The collected seeds were washed and dried at room temperature and stored in seed envelopes. For the experiment, a subset of pooled seeds (~100) was surface‐sterilized and germinated in standardized potting medium within climate‐controlled chambers maintained at 28°C–30°C, 68%–70% relative humidity and a 16:8 h light:dark photoperiod. Seedlings were allowed to reach a uniform developmental stage defined by the presence of six to eight fully expanded true leaves before the initiation of herbivory treatments. The experimental design followed a randomized block structure comprising three primary treatment groups: specialist chewing herbivory ( *M. sexta* ), generalist sap‐sucking herbivory ( *A. craccivora* ), and an undamaged control group. For each treatment, three biological replicates were established, with each replicate consisting of pooled leaf tissue from three to five individual plants to mitigate the impact of individual‐level randomness in gene expression (Sun et al. 2017).

Eggs of *M. sexta* were obtained from a commercial laboratory colony (Great Lakes Hornworm, MI, USA; Kariyat et al. 2012). Neonate larvae were reared on an artificial wheat‐germ‐based diet and allowed to reach the third instar before being transferred to the experimental plants. Two larvae were placed on the fourth and fifth fully expanded leaves of each plant and confined using clip cages, allowing continuous feeding for 48 h. This period was selected to capture the transition from immediate wounding responses to sustained secondary metabolite induction.

Adult cowpea aphids were collected from SLN plants in fields and then reared and maintained on the Black Beauty variety of *S. melongena* (Johnny's Selected Seed; Winslow, Maine). For the experiment, five nymphs of aphids were carefully transferred to the abaxial surface of target leaves using a fine‐tipped camel‐hair brush. The aphids were allowed to feed for 48 h, synchronized with the *M. sexta* treatment to allow for direct temporal comparison of the early transcriptomic profiles. Control plants were maintained under identical conditions, including the application of empty clip cages, to account for any potential gene expression changes induced by mechanical touch or cage‐induced microclimate alterations. After the 48‐h infestation period, insects were carefully removed, and leaf tissue was immediately harvested. To preserve the instantaneous state of the transcriptome, harvested tissues were flash‐frozen in liquid nitrogen within seconds of removal and stored in a cryogenic freezer at −80°C (Figure S1).

### 2.2 RNA Isolation and Sequencing

Total RNA was extracted using a commercially available RNA Plant Minikit (Qiagen), specifically designed for tissues rich in polyphenolics following the manufacturers protocol. The concentration and purity of the resulting RNA were initially assessed using a NanoDrop 2000 spectrophotometer. RNA integrity was quantitatively determined using the RNA Nano 6000 Assay Kit on an Agilent 2100 Bioanalyzer system (Sun et al. 2017). Sequencing was performed on the Illumina NovaSeq 6000 platform generating 150 bp paired‐end reads. Raw reads were quality‐assessed using FastQC (Andrews 2010), adapter trimming was performed using Trimmomatic (Bolger et al. 2014), and reads failing quality thresholds were excluded. For each biological sample, sequencing depth ranged between approximately 30 and 50 million paired‐end reads.

### 2.3 Quantification of Transcript Expression and Pre‐Processing

Cleaned paired‐end reads were mapped to the Trinity‐assembled transcriptome and transcript‐level abundance was estimated using kallisto (v0.51.1; Bray et al. 2016) and generated as raw count values. Transcript counts were summarized at the unigene level to produce a count matrix for downstream analyses. Unigenes were retained only if they exhibited a minimum expression level of at least one count per million (CPM) in at least three samples, corresponding to the number of biological replicates per treatment. Raw and normalized library sizes, normalization size factors, and per‐sample QC metrics are summarized in Table S1.

### 2.4 Transcriptome Reference Selection and Functional Annotation

In the absence of a fully annotated reference genome for *S. elaeagnifolium* , RNA‐seq data were analyzed using a de novo transcriptome‐based quantification approach (Raghavan et al. 2022; Brungardt and Bock 2023). Cleaned reads were quantified against a previously generated Trinity‐assembled transcriptome of *S. elaeagnifolium* and transcript abundance estimates were obtained for all samples based on this assembly (Haas et al. 2013). Transcript identifiers followed Trinity nomenclature (e.g., TRINITY_DN*xxxx*), reflecting the use of a de novo assembled transcript references rather than genome‐based gene models (Ward et al. 2012). Functional annotation of transcripts was performed using the Trinotate pipeline (v3.0.1), integrating BLASTX homology searches against Swiss‐Prot, Pfam domain detection via HMMER, eggNOG orthology assignments, KEGG pathway mapping and Gene Ontology (GO) term classification (Skidmore 7 AD; Griffth 2026; Thunders et al. 2017; Trinotate: Trinity Transcriptome Functional Annotation; Dalhousie University 2026).

### 2.5 Differential Gene Expression Analysis

Differential gene expression (DGE) analysis was performed to identify transcriptional changes associated with *M. sexta* and *A. craccivora* infestation (Rosati et al. 2024). Transcript abundance was quantified at the unigene level and the number of fragments mapped to each Trinity‐assembled sequence was summarized as raw count data (Haas et al. 2013). Raw counts were imported into the DESeq2 package (v1.50.2) in R for statistical analysis (Love et al. 2018). DESeq2 estimates gene‐wise dispersion parameters and log2 fold changes using an empirical Bayes shrinkage framework, which stabilizes variance estimates for lowly expressed or highly variable genes and improves the reliability of differential expression inference (Love, Anders, and Huber 2014; Love, Huber, and Anders 2014). All downstream analyses, including principal component analysis (PCA), heatmap visualization and co‐expression network construction, were performed on variance‐stabilizing transformed (VST) counts generated using the DESeq2 package in R Studio. This procedure pulls gene‐specific estimates towards a global trend, thereby stabilizing the log2Fold Change (log2*FC*) for genes with low counts or high variability, which significantly reduces the rate of false positives in high‐impact transcriptomic studies (Love et al., 2014b; Raithel et al. 2016).

### 2.6 Pairwise Treatment Comparisons and Statistical Thresholds

The differential expression analysis focused on three predefined pairwise contrasts: (i) Aphid vs. Control (A_vs_C), to capture transcriptional responses associated with sap‐sucking herbivory; (ii) Hornworm vs. Control (H_vs_C), to identify responses induced by chewing herbivory; and (iii) Aphid vs. Hornworm (A_vs_H), to compare transcriptional responses between the two herbivore feeding guilds. For each contrast, differential expression was assessed independently using the DESeq2 statistical framework. Genes were classified as differentially expressed if they satisfied two criteria: an adjusted *p* value of < 0.05 (false discovery rate, FDR) following Benjamini–Hochberg correction for multiple testing and an absolute log2 fold change ≥ 1.0 (log2FC), corresponding to a minimum two‐fold change in expression relative to the control condition (Liu et al. 2021). The resulting differentially expressed gene (DEG) sets were visualized using volcano plots to assess the global distribution of expression changes and Venn diagrams to identify genes shared across the herbivory treatments (Liu et al. 2021).

### 2.7 Functional Annotation and Enrichment Analysis

Functional annotation was performed to interpret DEGs and co‐expression network components using a consensus of multiple annotation sources. Given that *S. elaeagnifolium* is a non‐model organism, annotation was primarily driven by orthology‐based functional transfer, which provides a higher degree of precision than a standard BLAST‐based homology search by distinguishing between orthologs (Hernández‐Plaza et al. 2026). GO terms were used to classify transcripts across the Biological Process (BP), Molecular Function (MF), and Cellular Component (CC) domains, while KEGG annotations enabled mapping of transcripts to metabolic and signaling pathways. Functional enrichment analysis of DEG sets was conducted using the clusterProfiler (v4.18.4) package in R (Yu et al. 2012) and also using the STRING database (Szklarczyk et al. 2025), with statistical significance (FDR cutoff of < 0.05) assessed against a background of all expressed transcripts (Yu 2012). Enriched KEGG Orthology terms were further linked to KEGG pathways using the KEGGREST (v1.50.0) package and pathway‐level trends were inferred based on the predominant direction of associated DEGs (Tenenbaum et al. 2024; Muley 2025). Transcription factor‐annotated DEGs were identified by filtering the DEG lists against GO Molecular Function terms GO:0043565 (sequence‐specific DNA binding) and GO:0003700 (DNA‐binding transcription factor activity) from the Gene Ontology database (Carbon and Mungall 2026). GO molecular function enrichment analysis of TF‐annotated DEGs was performed using the clusterProfiler package, with Benjamini–Hochberg correction applied (adjusted *p* < 0.05).

### 2.8 Weighted Gene Co‐Expression Network Analysis

To characterize transcriptional regulation beyond pairwise differential expression, weighted gene co‐expression network analysis (WGCNA) was performed using the WGCNA (v1.73) package in R (Langfelder and Horvath 2008). The variance‐stabilized expression values derived from the DESeq2 pipeline were used as input for WGCNA to minimize heteroscedasticity and reduce the influence of extreme expression values (Love et al., 2014a). An unsigned co‐expression network was constructed. The soft‐thresholding power (β) was selected by evaluating a scale‐free topology model fit and mean connectivity across a range of β values (1–30; Li et al. 2020). A value of *β* = 8 was chosen as a biologically appropriate compromise that supported partial scale‐free topology while maintaining sufficient network connectivity (Zhang and Horvath 2005; Li et al. 2020; Figure S2). Adjacency matrices were transformed into TOM (Topological Overlap Matrix, a measure of shared network connectivity between gene pairs used to define modules) and gene modules were identified using average linkage hierarchical clustering coupled with dynamic tree cutting (Li et al. 2020; Peng et al. 2025). Module eigengenes were correlated with experimental traits (Control, Aphid and Hornworm) using Pearson correlation analysis. Modules showing significant positive or negative associations with a given herbivore treatment (*p* ≤ 0.05) were designated as herbivore responsive. For each module, kME (module membership, correlation between a gene's expression and its module eigengene) and GS (Gene Significance, correlation between individual gene expression and a treatment condition) threshold was set at the value corresponding to the inflection point of the kME frequency distribution, ensuring that only genes in the high‐density upper tail of the distribution representing the most tightly co‐expressed and treatment‐relevant genes were retained.

### 2.9 Hub Gene Integration and Integrative Functional Interpretation

To further refine hub gene identification, co‐expression networks were exported to Cytoscape (v3.10.4; Shannon et al. 2003; Su et al. 2014) and the CytoHubba plugin (Chin et al. 2014) was used to assess node centrality using the Maximal Clique Centrality (MCC, a network centrality measure of node connectivity within a co‐expression module) algorithm (Chin et al. 2014). Genes consistently ranked among the top candidates by both kME and MCC metrics were designated as hub genes (Tripathi et al. 2024; Yu et al. 2024; Zheng et al. 2025). To validate and contextualize hub gene functions, secondary enrichment analyses were performed using GO Biological Process and KEGG annotations, with all expressed genes serving as the background universe. These analyses were used exclusively to support functional interpretation of identified hubs rather than for de novo pathway discovery, ensuring consistency and comparability across herbivore feeding guilds.

## 3 Results

### 3.1 Sequencing Depth and Data Preprocessing

RNA‐seq libraries exhibited substantial variation in sequencing depth, with raw library sizes ranging from 7.4 to 59.9 million reads across samples (Figure S3). To account for differences in sequencing depth and RNA composition, count data were normalized prior to downstream analyses. Normalization effectively adjusted library sizes to a comparable scale across all samples (Figure S3). In total, expression was quantified for 37,285 transcripts, of which 35,777 were retained after pre‐filtering to remove lowly expressed genes. This filtering step eliminated approximately 4% of transcripts, indicating a conservative approach that minimized noise while retaining the majority of expressed genes for differential expression analysis.

### 3.2 Differentially Expressed Genes

**FIGURE 1:**
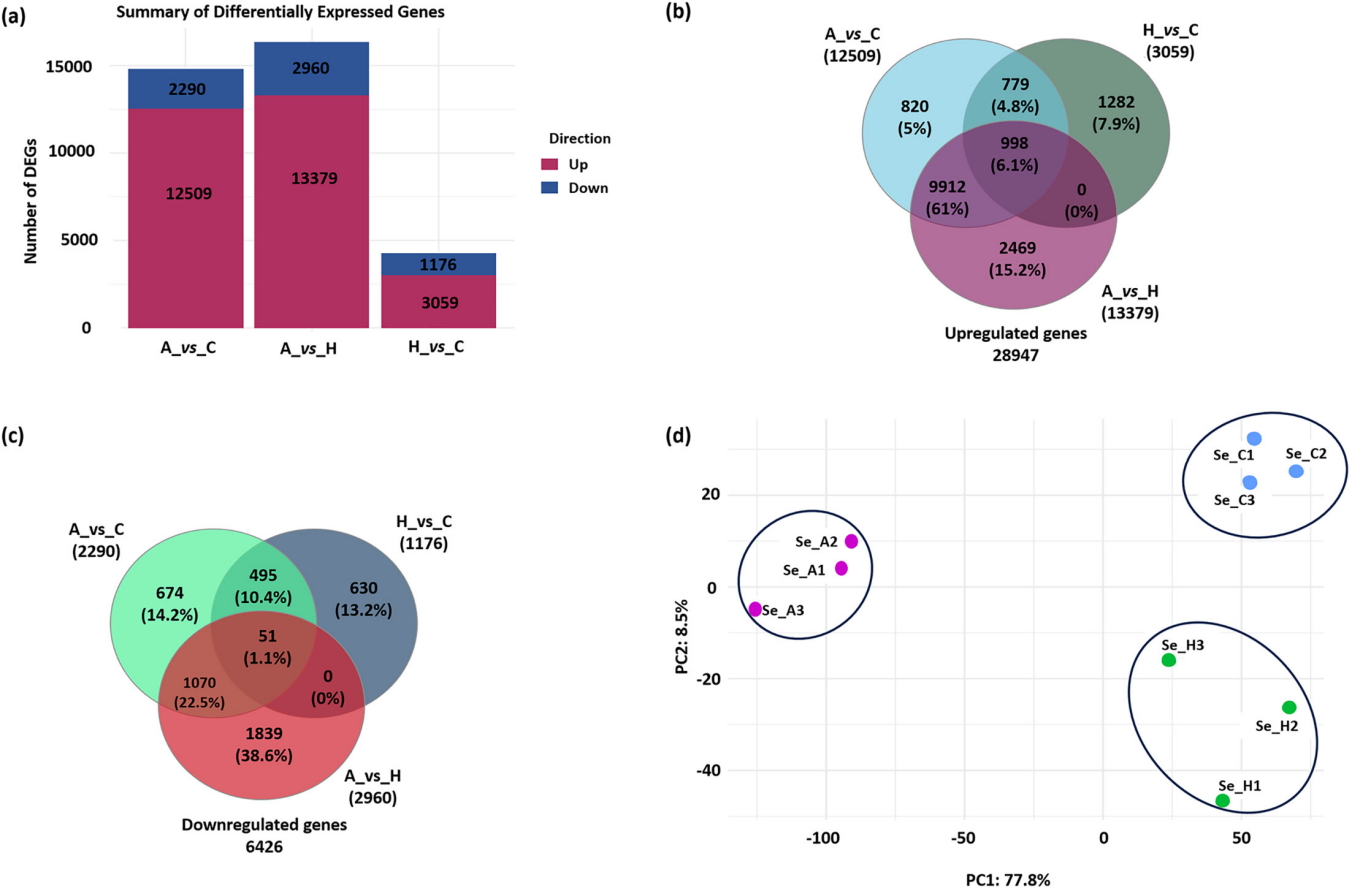
Exploratory data analysis of the *Solanum elaeagnifolium* transcriptome across herbivore treatments. (a) Stacked bar chart summarizing total DEG counts per comparison, partitioned by direction of regulation. (b) Venn diagram of upregulated DEGs (log_2_FC > 1, FDR < 0.05) across all three comparisons. (c) Venn diagram of downregulated DEGs (log_2_FC < −1, FDR < 0.05) across all three comparisons (comparison labels: A_vs_C = Aphid‐infested vs. Control; A_vs_H = Aphid‐infested vs. Hornworm‐infested; H_vs_C = Hornworm‐infested vs. Control). (d) Principal component analysis (PCA) of variance‐stabilizing transformed (VST) counts (samples: Aphid‐infested (Se_A1–Se_A3), Hornworm‐infested (Se_H1–Se_H3), and uninfested controls (Se_C1–Se_C3)).

Differential expression analysis revealed substantial transcriptional changes across all contrasts (Figure 1). Compared with the control, aphid infestation resulted in 14,799 DEGs (12,509 upregulated and 2290 downregulated), whereas hornworm infestation led to 4235 DEGs (3059 upregulated and 1176 downregulated; Figure 1a). Direct comparison between aphid and hornworm treatments identified 16,339 differentially expressed genes, comprising 13,379 upregulated and 2960 downregulated transcripts. Across all contrasts, upregulated genes predominated, and Venn analyses indicated both shared and contrast‐specific gene sets, reflecting distinct transcriptional responses to piercing‐sucking and chewing herbivory (Figure 1b,c). To evaluate whether these transcriptional differences were consistent across biological replicates and treatment groups, expression patterns were further examined using variance‐stabilized data. Variance‐stabilized transformation (VST) showed clear separation among treatments. Principal component analysis (PCA) revealed that the first principal component (PC1 = 77.8%) distinctly separated herbivore‐treated samples from controls, while the second component (PC2 = 8.50%) further distinguished aphid and hornworm treatments (Figure 1d).

Having established clear separation and reproducibility among treatments, we next examined the magnitude and direction of transcriptional changes at the individual gene level. Volcano plot analyses demonstrated extensive transcriptional reprogramming in SLN against aphid and hornworm infestation (Figure S4). Notably, aphid infestation induced a higher proportion of large‐magnitude transcriptional changes (85.8% of DEGs with log_2_FC > 1, median log_2_FC = 4.55) compared to hornworm attacks (74.6%, median log_2_FC = 3.89), suggesting that phloem‐feeding may trigger a more extreme transcriptional reprogramming relative to acute chewing damage.

### 3.3 Divergent Functional Enrichment Analysis

#### 3.3.1 GO Enrichment

**FIGURE 2:**
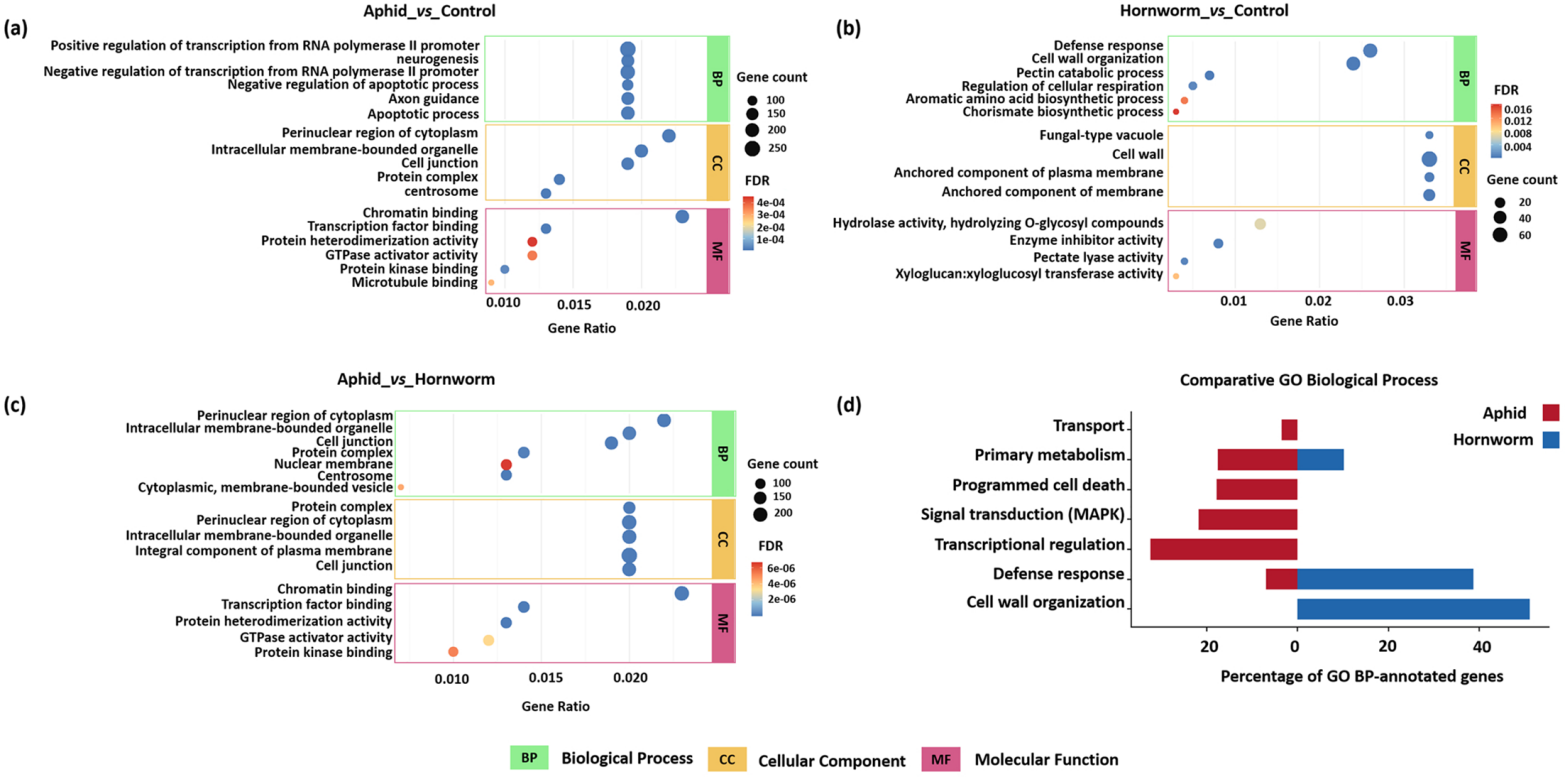
Gene ontology (GO) enrichment analysis of differentially expressed genes (DEGs) across three pairwise comparisons. All statistically significant DEGs from each comparison were used as input. Comparisons shown: (a) Aphid versus Control (14,799 DEGs; FDR < 0.05, log_2_FC > 1), (b) Hornworm versus Control (4235 DEGs; FDR < 0.05, log_2_FC > 1), (c) Aphid versus Hornworm (16,339 DEGs; FDR < 0.05, log_2_FC > 1), and (d) comparative bar chart of enriched GO Biological Process (BP) terms shared or contrasted between Aphid versus Control (blue) and Hornworm versus Control (red), expressed as the percentage of GO BP‐annotated genes (dot position indicates gene ratio, dot size represents gene count and colour denotes adjusted *p* value [FDR]. GO terms are grouped by ontology).

Functional characterization using GO confirmed distinct metabolic priorities for each feeding guild (Figure 2). GO‐based process quantification revealed distinct functional allocation patterns between treatments (Table S2). In cowpea aphid‐induced genes, transcriptional regulation (32%) and signal transduction (21%) processes accounted for a substantial proportion of enriched biological processes (Figure 2a). In contrast, hornworm‐induced genes showed a higher representation of cell wall organization (51%) and defense‐related processes (38%), particularly within the biological process and cellular component ontologies (Figure 2b). These contrasting GO profiles indicate herbivore‐specific functional responses rather than a generalized stress response. The differences in the aphid infestation predominantly eliciting metabolic related GO terms and hornworm infestation enriching defense associated terms (Figure 2d) indicate that silverleaf nightshade deploys herbivore‐specific functional programs rather than a uniform stress response across feeding guilds.

#### 3.3.2 Integrated KEGG Pathway and Orthology‐Based Functional Enrichment

**FIGURE 3:**
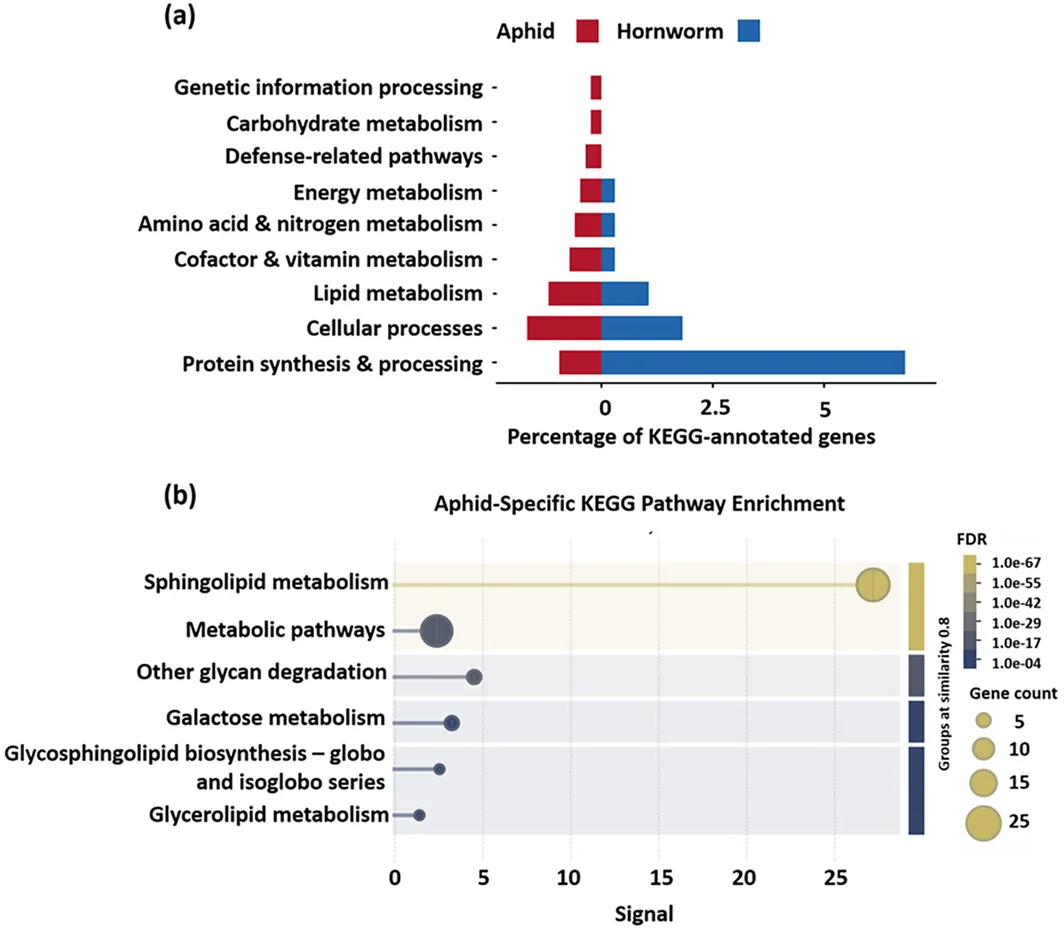
Comparative KEGG pathway enrichment analysis of herbivore‐induced differentially expressed genes (DEGs) in *Solanum elaeagnifolium* . DEGs were identified at FDR < 0.05 and log_2_FC > 1. (a) Horizontal bar chart showing the distribution of DEGs across broad KEGG functional categories for Aphid versus Control and Hornworm versus Control (The *x*‐axis represents the percentage of all KEGG‐annotated DEGs assigned to each functional category). (b) STRING network‐based KEGG pathway enrichment of Aphid‐specific DEGs (Aphid vs. Control; FDR < 0.05). Only pathways meeting enrichment significance in the Aphid versus Control comparison are shown; no equivalent pathways reached significance in the Hornworm vs. Control comparison (the *x*‐axis represents enrichment signal score derived from STRING functional enrichment analysis).

**FIGURE 4:**
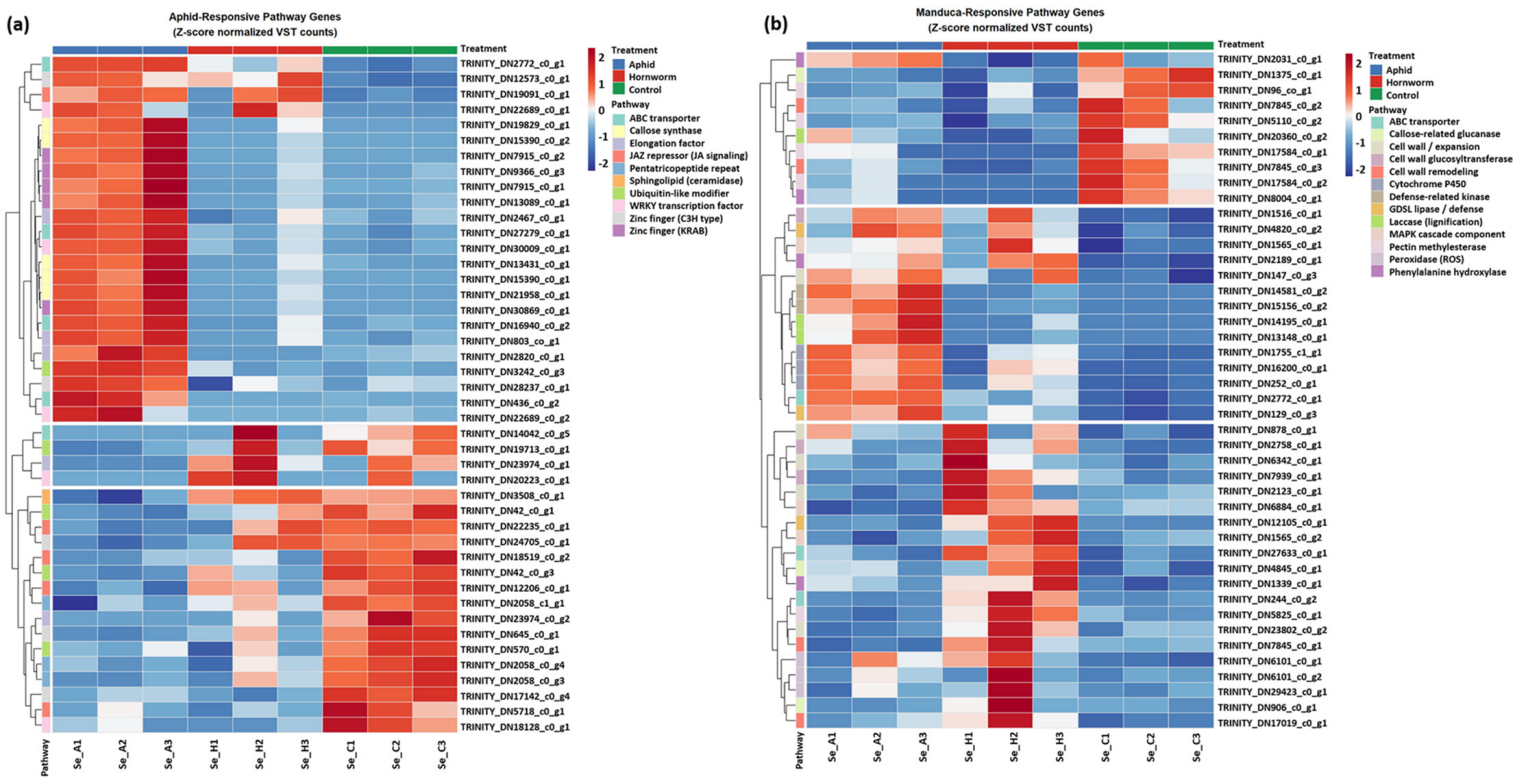
Heatmap of expression patterns of herbivore‐responsive pathway genes across all nine *Solanum elaeagnifolium* samples. (a) Rows represent individual genes annotated to key aphid‐associated pathways. (b) Rows represent individual genes annotated to key hornworm‐associated pathways; columns represent biological replicates grouped by treatment: Aphid (Se_A1‐A3, blue), Hornworm (Se_H1‐H3, red) and Control (Se_C1‐C3, green). Expression values are *Z*‐score normalized VST counts, where red indicates relatively high expression and blue indicates relatively low expression.

To place these functional patterns into a pathway‐level context, KEGG enrichment analysis was performed. Aphid infestation primarily enriched pathways related to central carbon metabolism, MAPK signalling, ABC transporters and sphingolipid metabolism, indicating metabolic reallocation and signalling adjustments associated with piercing–sucking herbivory (Figure 3a). These pathway‐level trends were supported at the gene level by enrichment of KEGG orthology terms involved in transcriptional regulation, cytoskeletal organization and protein modification, including zinc finger transcription factors, cofilin and glycosyltransferases, consistent with cellular and regulatory remodelling rather than a strong induction of specialized defense pathways (Table 1). In contrast, hornworm feeding resulted in enrichment of pathways associated with ribosome‐related protein synthesis and processing, cellular processes and core metabolic functions, reflecting extensive transcriptional and translational activity following chewing‐induced tissue damage (Table S3). Correspondingly, KO‐level enrichment highlighted genes involved in cell wall modification (e.g., xyloglucan:xyloglucosyl transferase and pectinesterase), calcium‐dependent signalling and transcriptional regulation, supporting structural and signalling responses characteristic of acute damage (Table 1). Several pathways were commonly enriched across both feeding guilds, including cellular processes, lipid metabolism, protein synthesis and processing, cofactor and vitamin metabolism, amino acid and nitrogen metabolism and energy metabolism, although with differing enrichment magnitudes (Figure 3a). Consistent with GO, KEGG pathway analysis of the aphid versus hornworm contrast revealed limited divergence at the metabolic pathway level, with cellular and lipid‐associated processes contributing the largest, although modest, proportions of differentially represented pathways. However, aphid‐responsive genes showed significant clustering in metabolic pathways (Figure 3b), hornworm‐responsive genes did not meet the enrichment threshold for specific metabolic pathways, suggesting a more diffuse or signalling‐based transcriptional response. However, the expression patterns of key pathway‐associated DEGs are visualised in Figure 4, which shows herbivore‐specific transcriptional clusters across all three comparisons. Gene IDs, fold changes, and significance values for all depicted genes are provided in Appendix S1.

**TABLE 1:** Comparative KEGG pathway enrichment of differentially expressed genes across three pairwise comparisons: Hornworm versus Control, Aphid versus Control, and Hornworm versus Aphid in *Solanum elaeagnifolium.*

KEGG ID	Description	Fold enrichment	*z* score	*p*	Count
Hornworm against Control					
K08235	Xyloglucan:xyloglucosyl transferase	8.10	6.91	0.00	7
K01051	Pectinesterase	6.94	5.78	0.00	6
K02979	Small subunit ribosomal protein S28e	4.96	4.56	0.00	6
K09338	Homeobox‐leucine zipper protein	3.65	3.56	0.00	6
K13412	Calcium‐dependent protein kinase	3.40	3.05	0.01	5
Aphid against Control					
K09228	KRAB domain‐containing zinc finger protein	1.52	2.24	0.02	15
K05765	Cofilin	1.74	2.31	0.02	9
K00710	Polypeptide *N*‐acetylgalactosaminyltransferase	1.59	1.94	0.05	9
Hornworm against Aphid					
K09228	KRAB domain‐containing zinc finger protein	1.59	2.77	0.00	17
K10380	Ankyrin‐1	1.69	2.62	0.01	12
K00710	Polypeptide *N*‐acetylgalactosaminyltransferase [EC:2.4.1.41]	1.81	2.83	0.00	11
K00966	Mannose‐1‐phosphate guanylyltransferase [EC:2.7.7.13]	1.77	2.48	0.01	9
K05679	ATP‐binding cassette, subfamily G (WHITE), member 1	1.77	2.48	0.01	9

*Note:* Entries represent the top enriched KEGG terms per comparison based on zScore ranking (*p* < 0.05). Enrichment analysis was performed using STRING functional enrichment with all expressed genes as background. Fold Enrichment: Ratio of observed gene frequency in the DEG list to expected frequency in the reference genome. *z* score: statistical measure of the distance of the observed gene count from the expected mean under the null hypothesis; higher values indicate stronger enrichment. *p* value: probability value for enrichment significance; values less than 0.05 were considered significant (*p* values reported as 0.00 indicate *p* < 0.001). Count: Number of differentially expressed genes mapped to the specific KEGG identifier.

**FIGURE 5:**
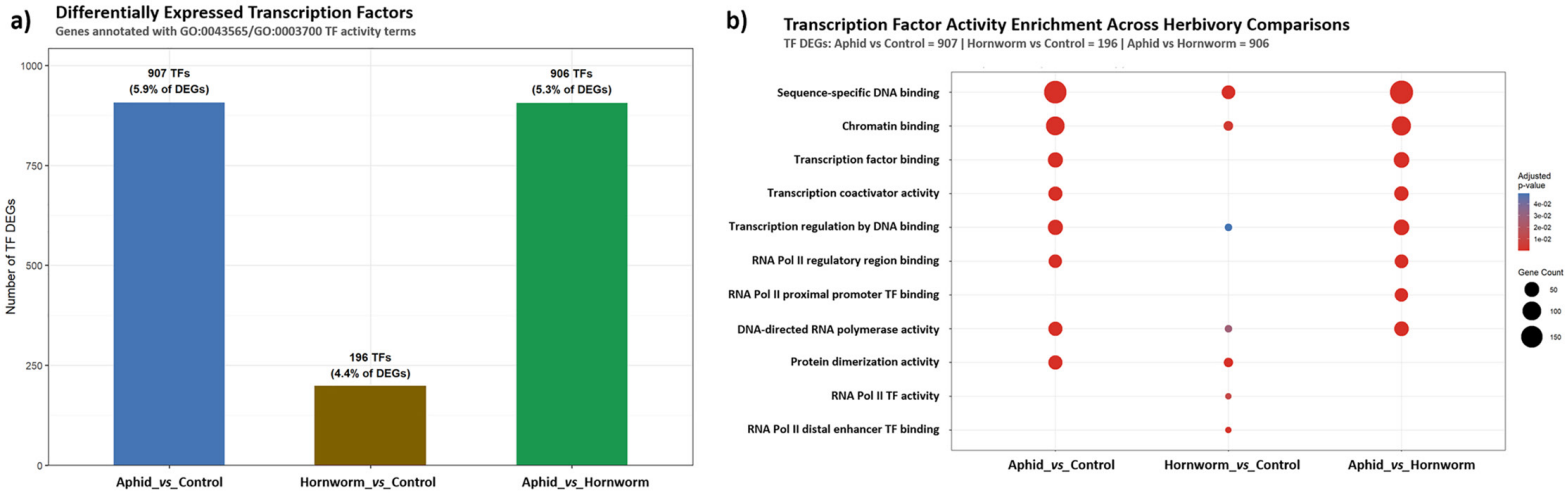
Transcription factor activity enrichment across herbivory comparisons. (a) Number of differentially expressed transcription factor genes per comparison, identified based on GO:0043565 and GO:0003700 annotations (Percentages indicate proportion of total DEGs per comparison), (b) Dot plot showing significantly enriched GO molecular function terms among TF‐annotated DEGs for Aphid versus Control, Hornworm versus Control, and Aphid versus Hornworm comparisons (dot size represents gene count; color indicates BH‐adjusted *p* value).

To characterize the regulatory architecture underlying herbivore‐specific responses, transcription factor enrichment analysis was performed on DEGs from each treatment comparison (Figure 5). Transcription factor (TF) enrichment analysis identified 907 and 196 TF‐annotated DEGs in aphid and hornworm comparisons respectively (Figure 5a). Aphid and hornworm‐responsive TF DEGs were predominantly enriched for sequence‐specific DNA binding and chromatin binding activities, with aphid interactions showing notably broader and more significant enrichment across all TF categories compared to hornworms (Figure 5b), further supporting the regulatory complexity of the plant response to phloem‐feeding herbivory.

#### 3.3.3 Weighted Gene Co‐Expression Network Analysis

**FIGURE 6:**
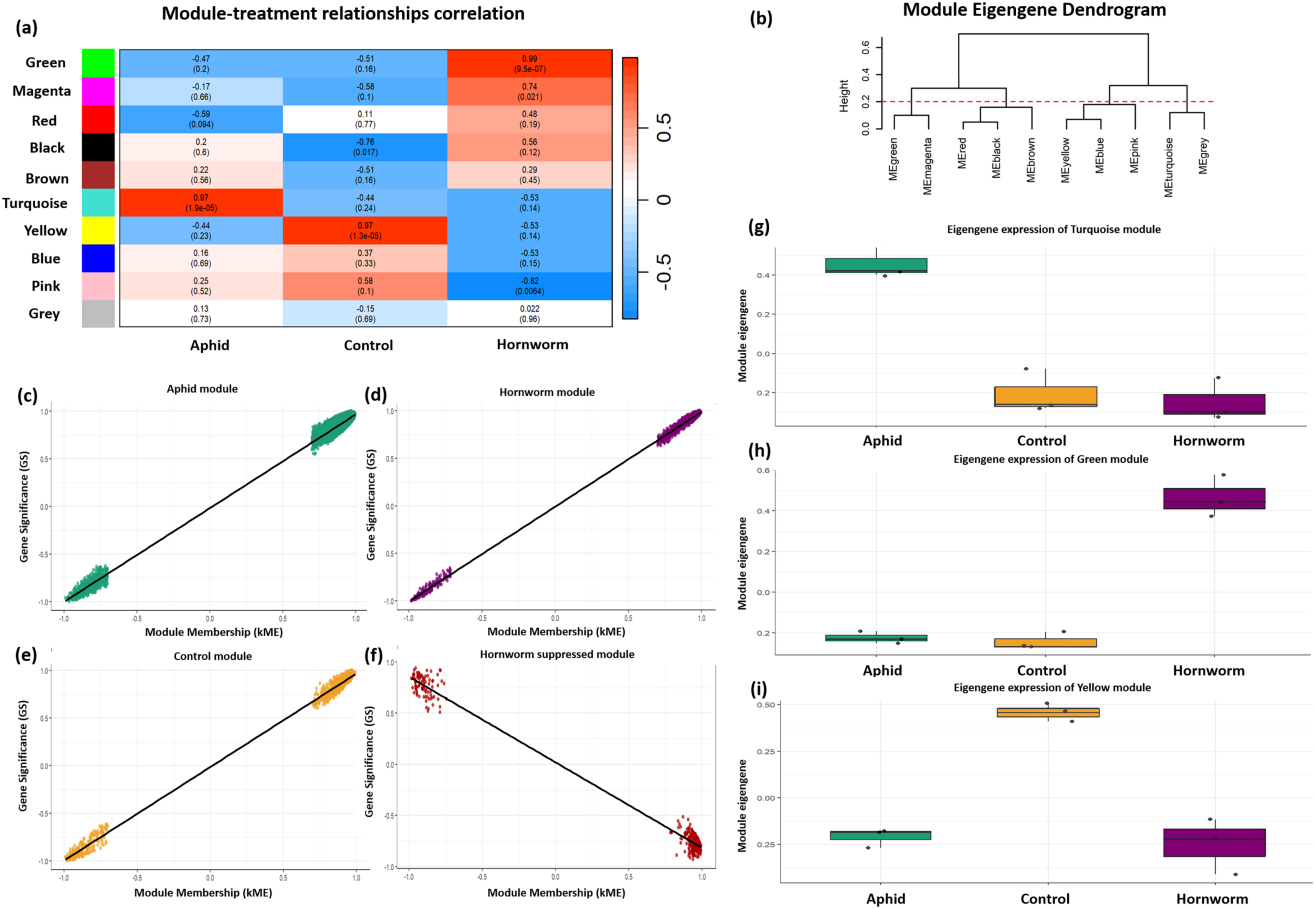
Weighted gene co‐expression network analysis (WGCNA) of the *Solanum elaeagnifolium* herbivore transcriptome. (a) Module‐trait relationship heatmap showing Pearson correlation coefficients between module eigengenes (rows) and treatment groups. Each cell displays the correlation value with the corresponding *p* value in parentheses. (b) Hierarchical clustering dendrogram of module eigengenes illustrating the relatedness of co‐expression modules. (c) Scatter plots of gene significance (GS; correlation of individual gene expression with treatment) versus Module Membership (kME; correlation of gene expression with module eigengene) for four representative modules: (c) Turquoise module‐Aphid‐specific; (d) Green module‐Hornworm‐specific; (e) Yellow module‐Control‐enriched; (f) Pink module‐genes suppressed under Hornworm treatment. (g) Boxplots of module eigengene (ME) expression values across treatment groups (Aphid, Control, Hornworm; *n* = 3 per group) for three biologically relevant modules: (g) Turquoise‐Aphid‐specific activation; (h) Green‐Hornworm‐specific activation; (i) Yellow‐elevated expression under Control conditions.

WGCNA was conducted using variance‐stabilized expression data to identify modules of co‐regulated genes across aphid, hornworm, and control treatments. Network construction and module detection resulted in the identification of 10 distinct modules (Appendix S2), excluding the grey module, which contained genes not assigned to any co‐expression cluster. Module sizes varied substantially, ranging from 143 genes in the magenta module to 18,289 genes in the turquoise module, indicating both tightly and broadly co‐expressed gene sets (Table S4). Hierarchical clustering of module eigengenes revealed clear separation among modules, with limited eigengene merging at the selected cut height, supporting the stability of the detected co‐expression structure (Figure 6a). The module eigengene dendrogram further indicated that major modules remained distinct, suggesting minimal redundancy among co‐expression patterns (Figure 6b).

To assess the association between gene co‐expression modules and experimental treatments, module eigengenes were correlated with aphid, hornworm, and control conditions. Several modules showed strong and statistically significant correlations with specific treatments. The turquoise module exhibited a strong positive correlation with aphid infestation (*r* = 0.97, *p* = 1.9 × 10^−5^) and a negative correlation with hornworm treatment (*r* = −0.53). In contrast, the yellow module showed a strong positive correlation with control samples (*r* = 0.97, *p* = 1.3 × 10^−5^) and negative correlations with both herbivore treatments. The green module displayed a strong positive correlation with hornworm infestation (*r* = 0.99, *p* = 9.5 × 10^−7^), while showing negative correlations with aphid and control conditions (Figure 6a).

The relationship between GS and kME was further examined for key treatment‐associated modules (Figure 6). Scatter plots of gene significance versus module eigengene connectivity demonstrated strong linear relationships for aphid, hornworm, and control‐associated modules, indicating that genes most strongly correlated with treatments also showed high module membership (Figure 6c–e). Conversely, the hornworm‐suppressed module displayed an inverse relationship between gene significance and module membership, consistent with coordinated downregulation within this module (Figure 6f). Module eigengene expression patterns across treatments further supported the module–trait correlation results (Figure 6g–i). The turquoise module showed elevated eigengene expression in aphid‐infested samples relative to control and hornworm treatments (Figure 6g), whereas the green module exhibited higher eigengene expression in hornworm‐infested samples (Figure 6h). The yellow module displayed higher eigengene expression in control samples compared with herbivore treatments, consistent with its strong association with the control condition (Figure 6i).

#### 3.3.4 Hub Gene Integration and Comparative Functional Interpretation

Hub genes were identified from treatment‐associated WGCNA modules based on high intramodular connectivity and strong correlations with experimental conditions. Distinct sets of hub genes were obtained for aphid and hornworm‐associated modules, with minimal overlap between treatments, indicating condition‐specific co‐expression architecture. A total of 19,746 hub genes were detected, with aphid‐associated hubs comprising the majority (17,684 genes), followed by hornworm (960), control (795), and hornworm‐suppressed hubs (307), indicating strong treatment‐dependent restructuring of network architecture (Table S4). Initial application of a uniform threshold (kME ≥ 0.80, GS ≥ 0.60) yielded an unacceptably large hub gene pool for the Turquoise module due to the characteristically tight and high‐density kME distribution of this module, where the majority of genes cluster near kME = 1.0 (Figure S5). To ensure biologically meaningful hub prioritization, module‐specific thresholds were therefore applied based on the inflection point of each module's intramodular kME frequency distribution: Aphid module‐kME ≥ 0.98 and GS ≥ 0.92; Hornworm and control module‐kME ≥ 0.95 and GS ≥ 0.90. These stringent, empirically derived thresholds ensured that only genes occupying the uppermost connectivity tier within each module representing the most tightly co‐expressed and treatment‐relevant hub candidates were retained for downstream functional analysis. Following application of module‐specific thresholds, the final hub gene set was refined to 535 genes (Aphid), 159 genes (Hornworm), and 97 genes (Control), representing a 96% reduction from the initial hub pool of 19,746 genes (Figure S5b).

Comparative eggNOG classification of hub genes identified within trait‐associated co‐expression modules revealed both shared and treatment‐preferential functional categories between aphid and hornworm infestations (Figure S6). Several eggNOG categories were common to both treatments, including serine/threonine protein kinases (COG0515), ankyrin repeat proteins (COG0666), leucine‐rich repeat proteins (COG4886), and cytochrome P450s (COG2124), indicating conserved involvement of signalling, protein–protein interaction, and detoxification‐related functions in herbivore‐responsive networks. Notably, these shared categories exhibited higher MCC scores in aphid‐associated networks than in hornworm‐associated networks (Table 2). Categories including zinc finger proteins (COG5048), GTP‐binding proteins (COG1100), calcium‐binding proteins (COG5126), helicases (COG0553), and protein tyrosine phosphatases (COG5599) showed consistently high MCC scores in aphid‐associated hubs. In contrast, hornworm‐associated hub genes were preferentially associated with functional categories linked to enzymatic activity and transport, including peptidases of the S8/S53 family (COG1404), ABC transporters (COG1131), dehydrogenases (COG1012), phosphatases (COG0631), and other transporter‐related functions (COG3104), showing comparatively lower MCC scores (Table 2).

**TABLE 2:** Comparative eggNOG‐based functional classification of WGCNA hub genes from aphid‐associated (Turquoise) and hornworm‐associated (Green) co‐expression modules in *Solanum elaeagnifolium* .

Shared eggNOG functional categories among aphid and hornworm associated hub genes			
Category	Description	MCC score^a^	
		Aphid	Hornworm
COG0515	Serine threonine protein kinase	78	43
COG0666	Ankyrin repeat	51	11
COG4886	Leucine rich repeat	38	14
COG2124	Cytochrome p450	27	12

Category	Description	MCC score^a^
eggNOG functional categories preferentially represented among aphid‐associated hub genes		
COG1100	GTP‐binding protein	39
COG5126	Calcium‐binding protein	32
COG5069	Microtubule associated monoxygenase, calponin and LIM domain containing	28
COG0553	Helicase	25
COG5599	Protein tyrosine phosphatase	24
eggNOG functional categories preferentially represented among hornworm‐associated hub genes		
COG1131	(ABC) transporter	11
COG1012	Dehydrogenase	10
COG0631	Phosphatase	10
COG3104	Transporter	9
ENOG41121N2	Zinc ion binding	9

^a^ Scores represents the Maximum Clique Centrality (MCC) value calculated using the cytoHubba plugin in Cytoscape, reflecting the topological importance of each gene within the co‐expression network. Higher MCC values indicate greater hub connectivity.

**FIGURE 7:**
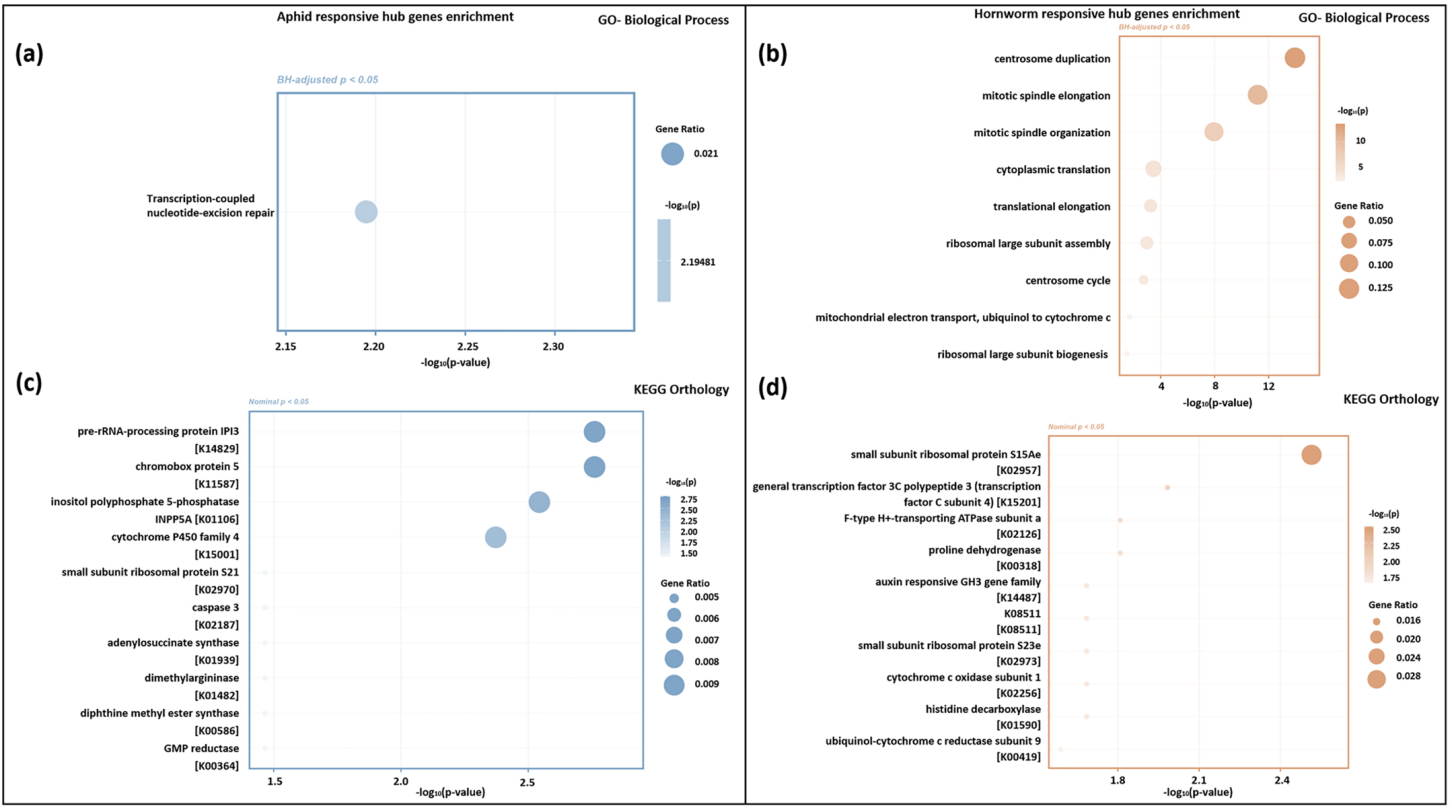
Functional enrichment of WGCNA‐identified hub genes in response to aphid and hornworm herbivory. Dot plots showing gene ontology (GO) biological process (panels a, b) and KEGG ortholog (KO) enrichment (panels c, d) for hub genes identified from co‐expression modules significantly associated with aphid (left column, blue) and hornworm (right column, orange) treatments. Each dot represents an enriched term; dot size reflects the gene ratio (proportion of hub genes annotated to the term) and dot color indicates statistical significance (−log_10_ *p* value; darker = more significant).

Initial hub gene candidates identified solely by module membership (kME ≥ 0.80, GS ≥ 0.60) yielded large gene sets with broad functional enrichment to reflect herbivory responses (Figure S7). However, functional enrichment of these filtered hub genes revealed treatment‐specific biological signatures that directly support the mechanistic distinction between aphid and hornworm responses (Figure 7). Aphid responsive hub genes showed highly specific functional enrichment, with GO biological process analysis identifying transcription‐coupled nucleotide‐excision repair as the sole significant term (Figure 7a). KEGG ortholog enrichment revealed additional functional diversity including chromatin organization, phosphoinositide signaling, apoptosis (programmed cell death), and cytochrome P450‐mediated detoxification, suggesting a coordinated epigenetic and stress‐survival response to aphid feeding (Figure 7b). Hornworm‐induced hub genes were significantly enriched for cell division and translational processes, including centrosome duplication, mitotic spindle organization, and ribosomal large subunit assembly (Figure 7c). KEGG ortholog analysis further identified ribosomal proteins, mitochondrial ATP synthase, and cytochrome oxidase, consistent with an energy‐intensive tissue repair and protein synthesis program following chewing herbivory (Figure 7d). Control hub genes were enriched for basal physiological processes including cellular response to water deprivation, hormone signaling, and lipid metabolic processes, alongside KEGG orthologs involved in lipid sensing, aldehyde detoxification, and membrane transport, reflecting constitutive homeostatic gene regulation in the absence of herbivory (Figure S8).

## 4 Discussion

**FIGURE 8:**
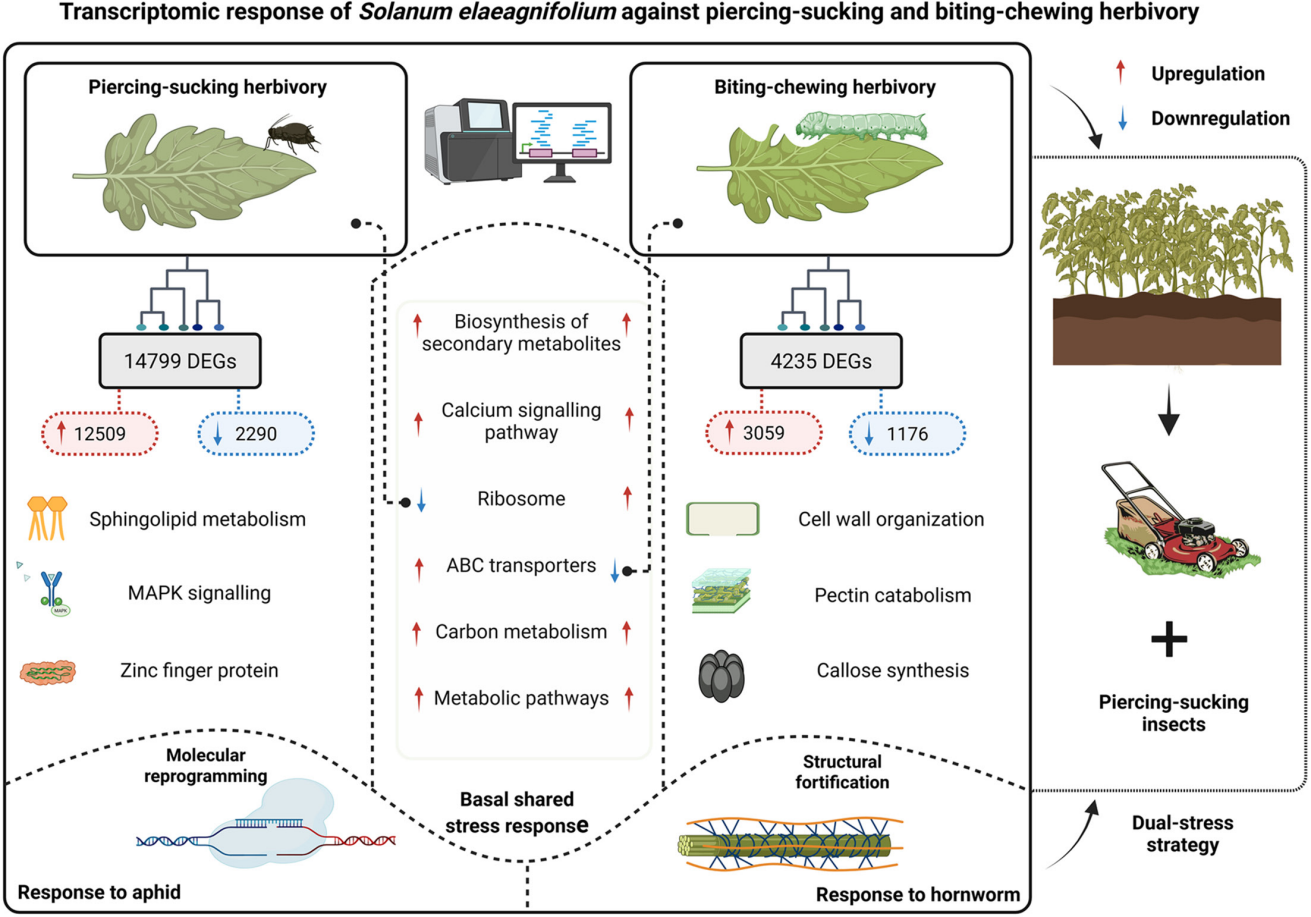
Conceptual model illustrating the transcriptional response of *S. elaeagnifolium* to piercing‐sucking ( *A. craccivora* ) and chewing ( *M. sexta* ) herbivory stress could lead to more effective biocontrol strategies that overcome the weed's plastic defensive mechanism (illustration created using BioRender).

The evolution of plant defense against herbivores is shaped by the constant pressure from diverse feeding guilds, each necessitating both a distinct physiological and molecular reprogramming. While these evolutionary pressures affect all vegetation, weeds represent the peak of such adaptive resilience, often outcompeting crops through superior defensive plasticity. In the context of SLN, a highly resilient and invasive weed, the ability to distinguish between and respond to varied biotic stressors is central to its ecological success (Tataridas et al. 2023). Our results provide the first molecular‐level evidence that SLN deploys distinct, guild‐specific transcriptional programs rather than a uniform defense response. Based on the comprehensive research carried out on ecological impacts of this weed, we addressed this gap using RNAseq analysis to characterize the divergent strategies SLN employs to adapt to guild‐specific herbivory. By moving beyond simple gene lists toward a systems‐level interpretation, we demonstrate that SLN does not deploy a uniform stress response but rather a highly specialized, herbivore‐dependent early transcriptomic reprogramming (Figure 8).

The considerably larger number of DEGs in response to aphid infestation compared to hornworm damage (14,799 vs. 4235 DEGs) needs to be considered carefully, particularly given that *M. sexta* is a Solanaceae specialist with well‐documented co‐evolutionary adaptations to host defenses. Guild‐dependent reprogramming parallels findings in *Populus tremula* , where sucking and chewing herbivores triggered largely non‐overlapping transcriptomic and metabolomic responses (Pastierovič et al. 2024), reinforcing the generality of guild‐specific defense allocation across distantly related plant taxa.

In the current study, we hypothesize that this difference reflects complementary mechanisms. Primarily, *M. sexta* is known to suppress broad‐spectrum plant transcriptional responses through oral secretions containing glucose oxidase and other salivary effectors that reduce jasmonate‐mediated defenses (Musser et al. 2012; Heidel‐Fischer and Vogel 2015), potentially limiting the transcriptome‐wide response despite causing significant tissue damage. Second, SLN is an invasive weed with no co‐evolutionary history with aphid species in its introduced range and novel herbivore associations are documented to elicit broader, less targeted transcriptional responses compared to specialist interactions (Züst and Agrawal 2016).

This asymmetry may also reflect fundamental differences in feeding duration and damage continuity. Aphid feeding is sustained and minimally destructive, requiring the plant to maintain continuous surveillance of phloem integrity and osmotic status over the full 48‐h window, which likely involves a broader set of regulatory and signalling genes over time. Chewing damage by *M. sexta* , by contrast, is acute and spatially localized, triggering a rapid, targeted wound‐response transcriptional burst that periods once structural and defense genes are induced, without requiring sustained systemic surveillance. This distinction parallels reports in Arabidopsis and other Solanaceae, where persistent phloem‐feeders elicit broader transcriptional reconfiguration than short‐duration mechanical damage (Appel et al. 2014; Walling 2008).

Consistent with this framework, the clear separation of treatments in PCA (PC1 = 77.80%; Figure 1d) supports the interpretation that the plant response is both robust and specific to the feeding stimulus. The use of field‐collected seeds might have the possibility of transgenerational epigenetic priming, whereby parental herbivory experience may influence offspring defense gene expression (Holeski et al. 2012; Rasmann et al. 2012). However, transgenerational priming in plants has been consistently shown to produce quantitative shifts in defense response magnitude, such as enhanced glucosinolate production or increased trichome density rather than qualitative reorganization of the transcriptome (Holeski et al. 2012; Luna et al. 2012; Slaughter et al. 2012).

Consistent with other solanaceous species, divergent feeding modes elicit largely non‐overlapping transcriptomic signatures (Hermsmeier et al. 2001; Ali and Agrawal 2012; Lortzing et al. 2017). The disproportionate enrichment of transcription factor activity terms in aphid‐responsive DEGs, which included nearly five‐fold more TF‐annotated genes than hornworm‐responsive DEGs, reinforces the interpretation that aphid feeding triggers extensive transcriptional recalibration rather than targeted biosynthetic activation. This regulatory response, resulting in the enrichment of chromatin binding and RNA Pol II‐associated TF activities (Figure 7), is consistent with a capacity for dynamic transcriptional reprogramming throughout prolonged aphid infestation, consistent with the established roles of chromatin‐mediated regulation and transcription factor networks in coordinating the plant defense responses (Pieterse et al. 2012; Ramirez‐Prado et al. 2018).

In *Arabidopsis thaliana* , studies show overlapping gene expression between sucking and chewing insects, representing conserved stress responses, while distinct DEGs highlight herbivore‐specific adaptations (Appel et al. 2014; Liu et al. 2022). In the current study, hornworm infestation induced a more acute response characterized by higher fold enrichments in gene expression (Table 1), a pattern typical of tissue loss associated with chewing herbivory. In contrast, aphid infestation elicited a broader, more distributed transcriptomic shift. This suggests that while hornworm damage triggers an intense and localized defense, aphid feeding involving subtle stylet penetration and the secretion of manipulative effectors may require a more complex and systemic modulation of host cellular processes for effective defense (Ali and Agrawal 2012).

This distinction is consistent with the established paradigm where chewing insects primarily activate jasmonate‐mediated structural and chemical defenses, while piercing‐sucking insects engage in complex molecular signalling interference and metabolic hijacking (Walling 2008; López‐Carretero et al. 2018). In the present study, observed transcriptional signatures include cell wall organization and callose synthesis under hornworm damage, whereas broad metabolic reprogramming including sphingolipid metabolism and MAPK signalling under aphid infestation aligns with this framework at the transcriptomic level, though direct phytohormone quantification would be required to confirm pathway activation.

To understand these divergent responses functionally, the integration of GO, KEGG and eggNOG findings revealed a clear prioritization of biological processes that differs by damage type. For hornworm, *M. sexta* , the enrichment was heavily biased toward cell wall organization and defense‐related processes (Table S2): specifically focusing on structural reinforcement through cellulose and pectin modification, but also including rapid synthesis, processing and mobilization of defense‐related compounds during chewing herbivory (Howe and Jander 2008; War et al. 2012). Enrichment of enzymatic and transport‐related eggNOG categories (Table 2) among hornworm‐associated hubs is consistent with established plant defense strategies against lepidopteran larvae, involving protein turnover, secondary metabolite deployment and detoxification (Zhu‐Salzman and Zeng 2015). Similar patterns have been reported in *A. thaliana* , where caterpillar feeding led to the upregulation of defense‐related proteins following mechanical tissue damage (Appel et al. 2014).

This pattern is consistent with an allocation of resources toward physical barriers to limit further damage under chewing herbivore pressure, as documented in earlier studies (Vaahtera et al. 2019; Perez‐Alonso et al. 2025). The limited functional enrichment observed among hornworm‐suppressed hubs further suggests that chewing herbivory predominantly activates defense‐related networks rather than broadly represses core metabolic pathways.

Aphid‐responsive genes, by contrast, were significantly enriched in transcriptional regulation and signal transduction (Table S2) alongside ABC transporters, without enrichment of cell wall‐associated pathways. This response pattern differs from that reported in *A. thaliana* , where infestation by *Myzus persicae* also triggered regulation of cell wall‐related processes similar to those observed during lepidopteran attack (Appel et al. 2014). This indicates the possibility that SLN's response to aphids is not primarily structural but regulatory. It is because the emphasis on sphingolipid metabolism (Figure 5b) and carbohydrate reallocation suggests that aphids induce a shift in the plant primary metabolism. Interestingly, sphingolipids are known to function as signaling molecules that shape cellular responses (Begum et al. 2016), indicating a compensatory metabolic adjustment, potentially linked to aphid‐induced nutrient sinks altering host resource allocation (Goggin 2007; Kuśnierczyk et al. 2008).

Extending our pathway‐level insights to a systems perspective, WGCNA revealed discrete co‐expression modules: the aphid‐correlated turquoise module and hornworm‐correlated green module that orchestrated SLN's guild‐specific defenses into higher‐order functional networks. The high correlation between gene significance and module membership suggested that the most critical genes in the SLN‐herbivore interaction are not acting in isolation but are part of a synchronized regulatory network. In this context, the modular architecture allows the plant to fine‐tune specific metabolic sectors such as cell wall modification or transcriptomic plasticity observed in our initial DEG analysis. The identification of 19,746 hub genes emphasized the complexity of the SLN regulatory landscape, yet the functional divergence between these hubs is striking.

Aphid‐induced hub genes are consistent with a stress‐repair signature centered on DNA integrity and chromatin remodeling, while hornworm‐induced hub genes point toward cellular proliferation and energy metabolism, a divergence broadly mirroring the mechanistic distinction between phloem‐feeding and chewing herbivory (Figure 7). This pattern is consistent with the hypothesis that SLN may recalibrate internal signalling to counter aphid attacks through targeted adjustments to chromatin architecture and DNA integrity consistent with the sustained nature of phloem‐feeding herbivory (Züst and Agrawal 2016). Hornworm‐associated hub genes, in contrast, show enrichment for ribosomal biogenesis, mitochondrial respiratory chain components and the auxin‐responsive GH3 gene family, raising the possibility of crosstalk between auxin and jasmonate signaling, since AtGH3 family members can catalyze formation of the bioactive jasmonoyl‐isoleucine (JA‐Ile; Khan and Stone 2007). However, confirmation of the specific GH3 ortholog is required before inferring direct involvement in JA‐Ile biosynthesis. This may help explain, in part, why SLN mounts an efficient response to hornworms (Chavana et al. 2021; Vasquez et al. 2024), although they are specialised to feed on Solanaceous species (Kariyat et al. 2012).

Damage caused by hornworm feeding and the associated transcriptomic changes resembles responses reported during mechanical mowing in other plant systems (Dombrowski et al. 2020; Leclerc et al. 2024). Notably, mowing is the most common anthropogenic pressure SLN faces. Although the plants used in the current study were not subjected to mowing, we hypothesize that frequent mowing in their source environments may have exerted transgenerational selective pressures, resulting in an SLN population genetically primed for mechanical repair, and thus preadapted to chewing‐insect attacks. Transgenerational effects of this kind have been reported previously, where repeated wounding from herbivory or mowing led to heritable shifts in plant defense responsiveness and stress‐adaptive traits (Wang et al. 2016; Chavana et al. 2021; Gautam and Kariyat 2025; Shafi and Kariyat 2025).

Under this hypothesis, the plant may perceive chewing herbivory less as a novel threat than as a variant of mechanical tissue damage, which could explain the rapid activation of a conserved regrowth and reinforcement module (the Green WGCNA module) that seals damage and reinforces remaining tissue. This response would be expected to mitigate the impact of chewing insect infestation without excessive depletion of metabolic reserves. The response to *A. craccivora* , by contrast, appears to reflect a different strategy: extensive molecular reprogramming rather than reinforcement of physical barriers.

Consistent with this, nearly 90% of the identified aphid‐associated hub genes (the turquoise WGCNA module; Table S4) are annotated to signaling pathways and programmed cell death rather than structural defenses. This suggests the aphid‐associated response is oriented more toward signaling and resource regulation than toward physical defense. The large number of DEGs observed under aphid infestation suggests a broad and energetically costly transcriptional reprogramming. This is consistent with the hypothesis that aphid feeding disrupts host signalling networks and redirects metabolic resources, a pattern analogous to what has been described as molecular interference in other plant‐aphid interactions (Walling 2008; Züst and Agrawal 2016).

These findings suggest that SLN possesses an exceptionally plastic transcriptome, which may contribute to its status as a highly successful invasive weed (Petanidou et al. 2018). The capacity to mount a signalling‐heavy response to aphids while maintaining a distinct, high‐intensity structural response to chewing insects is consistent with a multi‐herbivore defense strategy involving guild‐specific resource allocation. This raises the possibility of a trade‐off in which SLN may not simultaneously optimize defenses against mechanical damage and metabolic drain, consistent with the contrasting physiological changes known to differentially engage SA and JA‐mediated signalling in phloem‐feeding versus chewing herbivory (Koornneef and Pieterse 2008; Thaler et al. 2012).

Our data are consistent with SLN prioritizing structural defense responses to chewing herbivory, plausibly reflecting selection pressure from the immediate consequences of tissue loss. Under aphid infestation, the broader, more diffuse transcriptional response observed here raises the hypothesis that prolonged phloem‐feeding may impose a greater metabolic burden (Karssemeijer et al. 2020; Quijano‐Medina et al. 2023; Vasantha‐Srinivasan et al. 2025), a hypothesis that will require direct testing through metabolomic profiling and herbivore performance assays in future work. These results are best viewed as a hypothesis‐generating framework for future studies into the molecular basis of weed‐insect interactions and the potential for targeting these regulatory modules in biocontrol or crop resilience strategies.

One of the major management strategies against SLN is mechanical mowing, which our data suggests the plant is evolutionarily adapted to tolerate through rapid structural repairs. However, this adaptation may be its Achilles' heel to exploit vulnerability. We propose a dual‐stress strategy, since the plant is primed to defend against mechanical damage but exhibits a non‐specific, resource‐intensive transcriptional response to piercing‐sucking insects; a sequential approach could potentially overwhelm its plasticity. Mechanical mowing would force the weed to commit its energy reserves to structural repair and regrowth. Piercing–sucking insects are likely to preferentially colonize regenerating tissues, which often differ physiologically from undamaged plant parts. The presence of herbivores with distinct feeding strategies may therefore place combined pressures on SLN, potentially exposing limitations in its defense responses under complex biotic stress conditions.

## 5 Conclusions

Transcriptomic analysis of *S. elaeagnifolium* reveals a highly specialized defense strategy tailored to the specific challenges posed by divergent feeding guilds (Figure 8). We have demonstrated that the response to *A. craccivora* is characterized by a massive, systemic molecular reprogramming involving 14,799 DEGs and a dominant regulatory hub governed by including chromatin organization, phosphoinositide signaling, and programmed cell death. In contrast, *M. sexta* triggers enrichment for cell division and translational processes, including centrosome duplication, mitotic spindle organization, and the ribosomal large subunit. The high correlation between *M. sexta* infestation and structural repair pathways is likely an evolutionary byproduct of adaptation to mechanical stresses like mowing, suggesting that this invasive weed perceives chewing damage similar to the mechanical wounding. By mapping these specific regulatory switches, we propose a novel dual‐stress management framework. Exploiting the plant's metabolic trade‐offs, specifically its intense commitment to transcriptional reprogramming under aphid stress versus its structural focus under mechanical or chewing stress.

While this study provides a comprehensive map of the SLN transcriptomic landscape, it is not without constraints. First, in the absence of a high‐quality reference genome, our reliance on transcript‐level inference may overlook certain genomic complexities, such as gene duplication or isoform‐level complexity. Second, transcriptomic profiling was performed at a single 48‐h time point, which captures early guild‐specific responses but does not resolve the temporal dynamics of induction and recovery. Third, transcript abundance does not necessarily reflect protein‐level or metabolite‐level outcomes and functional confirmation of candidate hub genes (e.g., via qRT‐PCR, proteomics or metabolomics) remains an important next step.

Future studies should prioritize qRT‐PCR and biochemical validation of key hub gene candidates, particularly chromobox protein 5 (K11587) as an epigenetic regulator of aphid response and auxin responsive GH3‐family gene (K14487) as direct mediators of jasmonate conjugation in hornworm‐challenged tissues, to establish the functional links implied by the transcriptional network architecture described here. Furthermore, while the transcriptomic shifts are profound, they do not always correlate linearly with metabolic outputs. The next logical steps should integrate metabolomics and proteomics approaches, both to determine whether these transcriptional hub activities translate into increased concentrations of specialized metabolites, and to characterize aphid salivary effectors that may modulate these host responses.

## Author Contributions

R.K. and M.T. conceived and designed the study. R.K. conducted the greenhouse experiments and herbivory treatments. A.K. and J.J. performed the transcriptome sequencing, DESeq2 analysis, and visualizations. A.K. wrote the original draft. R.K., J.J., and M.T. reviewed and edited the initial and final drafts. All authors contributed to data interpretation and reviewed and approved the final manuscript.

## Funding

This project was supported by startup funds received by Rupesh Kariyat from the University of Texas Rio Grande Valley and the Division of Agriculture, University of Arkansas.

## Supporting information


**Table S1:** Sequencing library summary.
**Table S2:** Top Gene Ontology (GO) enriched biological processes in the herbivore‐induced transcriptome of *S. elaeagnifolium* based on differentially expressed genes from Aphid vs. Control and Hornworm vs. Control comparisons.
**Table S3:** Quantitative regulation of major KEGG pathways in Solanum elaeagnifolium in response to aphid and hornworm herbivory relative to uninfested controls (padj < 0.05, log2FC > 1).
**Table S4:** Distribution of WGCNA co‐expression modules and treatment‐associated hub gene sets identified in Solanum elaeagnifolium transcriptome‐wide network analysis.
**Figure S1:** Schematic overview of the experimental design used to generate herbivore‐challenged transcriptomes of Solanum elaeagnifolium (SLN). (Illustration created using BioRender).
**Figure S2:** Selection of soft‐thresholding power for WGCNA network construction. To construct a scale‐free co‐expression network, the soft‐thresholding power (β) was evaluated across a range of values (1–30) using the WGCNA R package. **(Left)** Scale‐free topology model fit, expressed as the signed *R* ^2^ (scale‐free fit index), plotted against soft‐thresholding power. Signed *R* ^2^ values reflect the fit of the network's connectivity distribution to a scale‐free topology; negative values indicate a negative correlation structure. A soft‐thresholding power of *β* = 12 was selected as it achieved a satisfactory scale‐free fit (signed *R* ^2^ ≈0.40) while maintaining reasonable mean network connectivity. (Right) Mean connectivity of the co‐expression network plotted against soft‐thresholding power, showing the expected monotonic decrease with increasing power. The selected power of *β* = 12 represents an optimal balance between scale‐free topology approximation and sufficient mean connectivity for robust module detection. This parameter was used for all subsequent WGCNA analyses (Figure 6).
**Figure S3:** Quality control analysis of the Solanum elaeagnifolium transcriptome across different herbivore treatments (a) raw library sizes, (b) post‐normalization distribution (Samples: Aphid‐infested (Se_A1–Se_A3), Hornworm‐infested (Se_H1–Se_H3), and uninfested controls (Se_C1–Se_C3)).
**Figure S4:** Volcano plots illustrating differential expression profiles across herbivore treatments (a) Aphid vs. Control, (b) Hornworm vs. Control, and (c) Aphid vs. Hornworm.
**Figure S5:** Aphid module kME and Gene Significance distributions and hub gene re‐filtering summary. (a) Frequency distributions of Module Membership (kME; left) and Gene Significance (GS; right) for all genes within the Aphid‐associated Turquoise module (*n* = 18,289 genes). Both distributions are heavily concentrated near values of 1.0, reflecting the tight co‐expression structure of this module and justifying the application of a stringent hub gene threshold (kME ≥ 0.98, GS ≥ 0.92) to distinguish genuinely central hub genes from the dense background population. (b) Bar chart comparing hub gene counts before (Original) and after (Filtered) application of module‐specific kME and GS thresholds for each treatment comparison. Module‐specific thresholds applied: Aphid‐ kME ≥ 0.98 and GS ≥ 0.92; Manduca‐ kME ≥ 0.95 and GS ≥ 0.90; Control‐ kME ≥ 0.95 and GS ≥ 0.90.
**Figure S6:** Gene co‐expression network analysis and identification of top hub genes based on eggNOG orthologous groups.
**Figure S7:** Functional enrichment of unfiltered WGCNA hub gene candidates. GO Biological Process and KEGG pathway enrichment of the initial hub gene sets prior to network‐based filtering (Aphid: 17,684 genes; Hornworm: 960). Hub genes were identified based on module membership (kME > 0.8) and gene significance (GS > 0.6). The broad enrichment terms observed (neurogenesis, transcription regulation, axon guidance in aphid; cell wall organization, pectin catabolism in hornworm) reflect the inclusion of weakly connected and biologically non‐specific genes. Following stringent filtering retaining only high‐confidence hub genes (kME > 0.9, GS > 0.8, top 10% intramodular connectivity), functionally coherent and treatment‐specific enrichment signatures were recovered (Figure 7).
**Figure S8:** Functional enrichment of WGCNA‐identified hub genes in response to control. Dot plots showing Gene Ontology (GO) Biological Process (a) and KEGG Ortholog (KO) enrichment (b) for hub genes identified from co‐expression modules significantly associated with control hub. Each dot represents an enriched term; dot size reflects the gene ratio (proportion of hub genes annotated to the term) and dot color indicates statistical significance.

**Appendix S1:** Supplementary Information File 1.

**Appendix S2:** Supplementary Information File 2.

**Appendix S3:** Supplementary Information File 3.

## Data Availability

The raw data supporting the findings of this study are provided within the article's Supplementary Information files. Additional data are available from the corresponding author upon reasonable request.
