# Supplementary material for: Silverleaf Nightshade ( Solanum elaeagnifolium ) Undergoes Guild‐Specific Transcriptomic Reprogramming Against Chewing Versus Piercing Sucking Herbivory
Source: Physiol Plant. 2026 Sep 24;178(5):e71128. doi: 10.1111/ppl.71128 (PMC13612146; doi:10.1111/ppl.71128)
Supplement: Supplementary file 1 [file PPL-178-e71128-s002.docx]

**Silverleaf nightshade (*Solanum elaeagnifolium*) undergoes guild-specific transcriptomic reprogramming against chewing versus piercing sucking herbivory**

Arunsaikumar Karrem^1^, Matthew Terry^2^, Jagdish Jaba^3^ and Rupesh Kariyat^1^

**Affiliations:**

^1^ Department of Entomology and Plant Pathology, University of Arkansas, Fayetteville, AR 72701

^2^ [School of Integrative Biological and Chemical Sciences, The University of Texas Rio Grande Valley, Edinburg, TX 78501](https://www.utrgv.edu/cos/schools-and-departments/integrative-biology-and-chemistry/)

^3^ International Crops Research Institute for the Semi-Arid Tropics, Hyderabad, Telangana, India, 502324.

Supplementary Table 1. Sequencing library summary

| Sample ID | Treatment | Raw Library Size (reads) | DESeq2 Normalized Size | Size Factor | QC Status |
| --- | --- | --- | --- | --- | --- |
| Se_A1 | Aphid | 15,879,306 | 20,247,484 | 1.275 | Pass |
| Se_A2 | Aphid | 28,787,977 | 23,735,587 | 0.824 | Pass |
| Se_A3 | Aphid | 18,392,653 | 36,168,000 | 1.966 | Pass |
| Se_H1 | Hornworm | 7,423,399 | 32,894,933 | 4.431 | Pass* |
| Se_H2 | Hornworm | 30,236,597 | 121,308,532 | 4.012 | Pass |
| Se_H3 | Hornworm | 28,519,962 | 11,031,477 | 0.387 | Pass |
| Se_C1 | Control | 27,971,765 | 12,521,968 | 0.448 | Pass |
| Se_C2 | Control | 59,880,922 | 17,844,578 | 0.298 | Pass |
| Se_C3 | Control | 22,210,739 | 12,338,327 | 0.556 | Pass |

*Se_H1 had lower raw sequencing depth relative to other samples. Cook's distance analysis confirmed zero genes were flagged as outliers attributable to this sample (maximum Cook's D = 0.956), and PCA demonstrated consistent clustering with Hornworm replicates.

Supplementary Table 2. Top Gene Ontology (GO) enriched biological processes in the herbivore-induced transcriptome of *S. elaeagnifolium* based on differentially expressed genes from Aphid *vs.* Control and Hornworm *vs.* Control comparisons.

| ***Process*** | ***Gene count^#^*** | ***Per cent**** | ***Comparison*** |
| --- | --- | --- | --- |
| Transcriptional regulation | 779 | 32.42 | Aphid *vs.* Control |
| Signal transduction (MAPK) | 522 | 21.72 |  |
| Programmed cell death | 428 | 17.81 |  |
| Primary metabolism | 422 | 17.56 |  |
| Defense response | 168 | 6.99 |  |
| Transport | 84 | 3.50 |  |
| Cell wall organization | 70 | 51.09 | Hornworm *vs.* Control |
| Defense response | 53 | 38.69 |  |
| Primary metabolism | 14 | 10.22 |  |

^#^Gene count represents the number of differentially expressed genes (DEGs) associated with the specific Gene Ontology (GO) term

*Per cent values represent the proportion of differentially expressed genes assigned to a given GO biological process relative to the total number of genes mapped to the same GO ontology category (BP) within each treatment.

Supplementary Table 3. Quantitative regulation of major KEGG pathways in *Solanum elaeagnifolium* in response to aphid and hornworm herbivory relative to uninfested controls (padj < 0.05, log₂FC > 1)

| **Treatment** | **Pathway** | **Description** | **Up_Kos** | **Down_Kos** | **Regulation** |
| --- | --- | --- | --- | --- | --- |
| Aphid | map01100 | Metabolic pathways | 22 | 2 | Up |
|  | map01110 | Biosynthesis of secondary metabolites | 13 | 0 | Up |
|  | map03010 | Ribosome | 12 | 44 | Down |
|  | map01200 | Carbon metabolism | 6 | 0 | Up |
|  | map01230 | Biosynthesis of amino acids | 5 | 0 | Up |
|  | map04120 | Ubiquitin mediated proteolysis | 5 | 1 | Up |
|  | map04310 | Wnt signalling pathway | 5 | 0 | Up |
|  | map01212 | Fatty acid metabolism | 4 | 0 | Up |
|  | map02010 | ABC transporters | 2 | 0 | Up |
|  | map04020 | Calcium signalling pathway | 3 | 0 | Up |
| Hornworm | map03010 | Ribosome | 45 | 1 | Up |
|  | map02010 | ABC transporters | 0 | 2 | Down |
|  | map01100 | Metabolic pathways | 12 | 3 | Up |
|  | map01110 | Biosynthesis of secondary metabolites | 7 | 2 | Up |
|  | map04120 | Ubiquitin mediated proteolysis | 5 | 0 | Up |
|  | map04146 | Peroxisome | 4 | 0 | Up |
|  | map01200 | Carbon metabolism | 3 | 1 | Up |
|  | map01212 | Fatty acid metabolism | 3 | 0 | Up |
|  | map04310 | Wnt signalling pathway | 3 | 0 | Up |
|  | map04020 | Calcium signalling pathway | 2 | 0 | Up |

Up_Kos: Number of significantly upregulated KEGG Orthologs (log₂FC > 1, padj < 0.05) mapped to each pathway.
Down_Kos: Number of significantly downregulated KEGG Orthologs (log₂FC < −1, padj < 0.05) mapped to each pathway.
Regulation: Overall pathway regulation inferred from the dominant direction of KO-level expression changes; pathways are classified as 'Up' when upregulated KOs exceed downregulated KOs, and 'Down' when the opposite is true.

Supplementary Table 4. Distribution of WGCNA co-expression modules and treatment-associated hub gene sets identified in *Solanum elaeagnifolium* transcriptome-wide network analysis.

| **Module colour** | **Number of Genes** | **Per cent** |
| --- | --- | --- |
| Black | 582 | 2.17 |
| Blue | 3635 | 13.55 |
| Brown | 1046 | 3.90 |
| Green | 928 | 3.46 |
| Grey | 13 | 0.05 |
| Magenta | 143 | 0.53 |
| Pink | 314 | 1.17 |
| Red | 850 | 3.17 |
| Turquoise | 18289 | 68.16 |
| Yellow | 1032 | 3.85 |
| Total | 26832 | 100 |
| *Hub-wise gene list* | | |
| **Hub** | **Number of Genes** | **Per cent** |
| Aphid | 17684 | 89.56 |
| Control | 795 | 4.03 |
| Hornworm | 960 | 4.86 |
| Hornworm suppressed | 307 | 1.55 |
| Total | 19746 | 100 |

Supplementary Figure 1. Schematic overview of the experimental design used to generate herbivore-challenged transcriptomes of *Solanum elaeagnifolium* (SLN). (Illustration created using BioRender)

Supplementary Figure 2. **Selection of soft-thresholding power for WGCNA network construction.** To construct a scale-free co-expression network, the soft-thresholding power (β) was evaluated across a range of values (1–30) using the WGCNA R package. **(Left)** Scale-free topology model fit, expressed as the signed R² (scale-free fit index), plotted against soft-thresholding power. Signed R² values reflect the fit of the network's connectivity distribution to a scale-free topology; negative values indicate a negative correlation structure. A soft-thresholding power of **β = 12** was selected as it achieved a satisfactory scale-free fit (signed R² ≈ 0.40) while maintaining reasonable mean network connectivity. **(Right)** Mean connectivity of the co-expression network plotted against soft-thresholding power, showing the expected monotonic decrease with increasing power. The selected power of β = 12 represents an optimal balance between scale-free topology approximation and sufficient mean connectivity for robust module detection. This parameter was used for all subsequent WGCNA analyses (Figure 6).

Supplementary Figure 3. Quality control analysis of the *Solanum elaeagnifolium* transcriptome across different herbivore treatments (a) raw library sizes, (b) post-normalization distribution (Samples: Aphid-infested (Se_A1–Se_A3), Hornworm-infested (Se_H1–Se_H3), and uninfested controls (Se_C1–Se_C3))

Supplementary Figure 4. Volcano plots illustrating differential expression profiles across herbivore treatments (a) Aphid vs. Control, (b) Hornworm vs. Control, and (c) Aphid vs. Hornworm

Supplementary Figure 5. Aphid module kME and Gene Significance distributions and hub gene re-filtering summary. (a) Frequency distributions of Module Membership (kME; left) and Gene Significance (GS; right) for all genes within the Aphid-associated Turquoise module (n = 18,289 genes). Both distributions are heavily concentrated near values of 1.0, reflecting the tight co-expression structure of this module and justifying the application of a stringent hub gene threshold (kME ≥ 0.98, GS ≥ 0.92) to distinguish genuinely central hub genes from the dense background population. (b) Bar chart comparing hub gene counts before (Original) and after (Filtered) application of module-specific kME and GS thresholds for each treatment comparison. Module-specific thresholds applied: Aphid- kME ≥ 0.98 and GS ≥ 0.92; Manduca- kME ≥ 0.95 and GS ≥ 0.90; Control- kME ≥ 0.95 and GS ≥ 0.90.

Supplementary Figure 6. Gene co-expression network analysis and identification of top hub genes based on eggNOG orthologous groups

Supplementary Figure 7. Functional enrichment of unfiltered WGCNA hub gene candidates. GO Biological Process and KEGG pathway enrichment of the initial hub gene sets prior to network-based filtering (Aphid: 17,684 genes; Hornworm: 960). Hub genes were identified based on module membership (kME > 0.8) and gene significance (GS > 0.6). The broad enrichment terms observed (neurogenesis, transcription regulation, axon guidance in aphid; cell wall organization, pectin catabolism in hornworm) reflect the inclusion of weakly connected and biologically non-specific genes. Following stringent filtering retaining only high-confidence hub genes (kME > 0.9, GS > 0.8, top 10% intramodular connectivity), functionally coherent and treatment-specific enrichment signatures were recovered (Figure 7).

Supplementary Figure 8. Functional enrichment of WGCNA-identified hub genes in response to control. Dot plots showing Gene Ontology (GO) Biological Process (a) and KEGG Ortholog (KO) enrichment (b) for hub genes identified from co-expression modules significantly associated with control hub. Each dot represents an enriched term; dot size reflects the gene ratio (proportion of hub genes annotated to the term) and dot color indicates statistical significance.
